# Lower Prevalence of Atopic Dermatitis and Allergic Sensitization among Children and Adolescents with a Two-Sided Migrant Background

**DOI:** 10.3390/ijerph13030265

**Published:** 2016-02-26

**Authors:** Sinja Alexandra Ernst, Roma Schmitz, Michael Thamm, Ute Ellert

**Affiliations:** 1 Leibniz Institute for Prevention Research and Epidemiology—BIPS, Achterstraße 30, 28359 Bremen, Germany; 2 Robert Koch Institute, General-Pape-Str. 62-66, 12101 Berlin, Germany; SchmitzR@rki.de (R.S.); ThammM@rki.de (M.T.); EllertU@rki.de (U.E.)

**Keywords:** allergies, atopic hypersensitivity, children, adolescents, transients and migrants, prevalence

## Abstract

In industrialized countries atopic diseases have been reported to be less likely in children and adolescents with a migrant background compared to non-migrants. This paper aimed at both examining and comparing prevalence of asthma, allergic rhinoconjunctivitis and atopic dermatitis and allergic sensitization to specific IgE antibodies in children and adolescents with and without a migrant background. Using data of the population-based German Health Interview and Examination Survey for children and adolescents (KiGGS; *n* = 17,450; 0–17 years), lifetime and 12-month prevalence of atopic diseases and point prevalence of 20 common allergic sensitizations were investigated among migrants compared to non-migrants. Multiple regression models were used to estimate the association of atopic disease and allergic sensitization with migrant background. In multivariate analyses with substantial adjustment we found atopic dermatitis about one-third less often (OR 0.73, 0.57–0.93) in participants with a two-sided migrant background. Statistically significant associations between allergic sensitizations and a two-sided migrant background remained for birch (OR 0.73, 0.58–0.90), soybean (OR 0.72, 0.54–0.96), peanut (OR 0.69, 0.53–0.90), rice (OR 0.64, 0.48–0.87), potato (OR 0.64, 0.48–0.85), and horse dander (OR 0.58, 0.40–0.85). Environmental factors and living conditions might be responsible for the observed differences.

## 1. Introduction

It is well known that the prevalence of atopic diseases, such as asthma, allergic rhinoconjunctivitis and atopic dermatitis has increased substantially over the last decades, particularly in affluent Western societies and, thus, have become an important health problem, especially among children and adolescents [1,2,3,4,5,6,7,8].

In industrialized countries atopic diseases have been reported to be less likely in children and adolescents with a migrant background compared to non-migrants. For instance, an international comparative study conducted by the WHO among school-aged children (11, 13 and 15 years) showed that children with a two-sided migrant background were less often affected by at least one allergy (27.4%) compared to non-migrants (38.9%) [9]. Whether a migrant background is also associated with a lower prevalence of sensitization to specific IgE antibodies is not clear.

The German National Health Interview and Examination Survey for children and adolescents (KiGGS) is based on a representative sample of children and adolescents aged 0–17 years. A migrant-specific approach was used. Thus, the survey offers the opportunity to investigate atopic diseases as well as allergic sensitization among those with a migrant background compared to those with no such background in a sample including children and adolescents with a migrant background corresponding to their percentage in the German population. The purpose of this paper was: (i) to examine and compare lifetime and 12-month prevalence of atopic diseases in children and adolescents with and without migrant background; (ii) to examine and compare point prevalence of sensitization to 20 common allergens in children and adolescents with and without migrant background; and (iii) to investigate the association between atopic diseases and sensitization status with migration status, adjusted for known determinants of atopic diseases.

## 2. Materials and Methods

### 2.1. Design and Study Population

KiGGS is a population based cross-sectional study (*n* = 17,640, response rate: 66.6%) conducted from 2003–2006 by the Robert Koch Institute (RKI) Berlin, Germany. The study aimed at collecting comprehensive health data from a representative sample of children and adolescents aged 0–17 years. The sampling frame was based on a systematic sample of 167 primary sample units (PSUs), which was drawn from an inventory of German communities. As a second step an equal number of addresses per birth cohort were randomly selected from local population registries within the selected PSUs. The participants and their parents were invited to their local PSUs. The survey involved self-administered questionnaires, medical examinations and physical tests, as well as a standardized, computer-assisted personal interview (CAPI) about diseases. Detailed information on study design and response is given elsewhere [10,11].

Data on physician-diagnosed asthma, allergic rhinoconjunctivitis, and atopic dermatitis of the participants were collected by interviewing the attendant parents in CAPIs with trained pediatricians. Blood and urine samples were collected during medical examinations if parents and/or participants agreed. Information such as socio-demographic characteristics, living conditions and living environment were collected via the self-administered questionnaires [12].

In order to obtain a number of children and adolescents with migrant background that corresponds to their percentage in the German population a migration-specific approach was used. Children and adolescents from families of non-German nationality were included disproportionately high in the sample (oversampling). Within the pilot phase a large majority of people with migrant backgrounds could not participate in the study due to language barriers. Therefore, in the field phase, a multiple stage model was used for those with language barriers, *i.e*., simplifying language in invitation letters and information material, and, to further increase participation within this subgroup, invitation letters, information material as well as questionnaires were translated into six languages (Turkish, Russian, Serbo-Croatian, Arabic, English and Vietnamese). Migrant-specific public relation actions were targeted specifically at the migrant population of the individual local study center [11,13].

KiGGS was approved by the Charité-Universitätsmedizin Berlin Ethics Committee (application number 101/2000). The study was funded by the German Federal Ministry of Health and the German Federal Ministry of Education and Research. Informed consent was obtained from all parents.

### 2.2. Data Collection and Analysis

The interview questions: “Did a physician ever diagnose asthma/allergic rhinocunjunctivitis/ atopic dermatitis?” lead to estimation of lifetime prevalence. If “yes”, “Did the disease occur in the last 12 month?” provided 12-month prevalence estimation. In this context several common terms for the different atopic diseases were named in the interviews and, if necessary, were explained by the pediatrician. Questionnaires provided information on demographic characteristics (age, sex), migrant background, birth order, at least one atopic disease of mother and/ or father (atopic parents), parents’ smoking status (mother and/or father), living environment (rural, urban), and living conditions (mold-infested rooms). As proposed by Winkler and Stolzenberg information on household income, education, and profession were used to operationalize socio-economic status (low, middle, high) [14,15].

To determine migration status information on nationality, country of birth, year of immigration of both parents and information on languages spoken at home was drawn from questionnaires [11]. Children and adolescents with a two-sided migrant background were: (i) those who immigrated themselves and at least one parent was not born in Germany or (ii) those whose parents immigrated to Germany or did not have German citizenship. Children and adolescents with a one-sided migrant background were defined as children and adolescents who had only one parent who immigrated to Germany and/or has no German citizenship [13].

Blood serum samples collected during the medical examination were analyzed for specific IgE antibodies to 20 common allergens among children and adolescents aged 3–17 years using the ImmunoCap^®^ test system of Phadia, now Thermo Fischer Scientific (Uppsala, Sweden). Participants were considered to be sensitized to a specific allergen if the specific IgE serum level was greater than or equal to 0.35 kU/L.

We estimated and compared lifetime and 12-month prevalence with 95% confidence intervals (95%-CI) of physician diagnosed atopic diseases among migrants and non-migrants (one-sided and two-sided migratant background). Differences between non-migrants and those with a one-sided as well as non-migrants and those with a two-sided migrant background were considered to be statistically significant if 95%-CIs did not overlap and *p* ≤ 0.05. In addition, we investigated the association of atopic diseases in the last 12-months as well as allergic sensitization and migration status using bivariate and adjusted logistic regression models that provided odds ratios (OR) with 95%-CIs. Associations that were statistically significant (*p* ≤ 0.05) in bivariate models were investigated in subsequent adjusted logistic regression models. Multivariate models were adjusted for sex, age, socio-economic status, mold-infested rooms, smoking mother and/or father, living environment, birth order and parental atopy. Breastfeeding and East/West German residence were not associated in bivariate models. Data were analyzed using Complex Samples Procedure in SPSS/PASW Statistics 18.0.3 (IBM Corp., Armonk, NY, USA). In order to ensure that estimates derived from the KiGGS study are representative at the national level, survey weights were applied throughout statistical analyses. Weights were applied to correct for age, sex, residence (Eastern/Western Germany) and nationality.

## 3. Results

### 3.1. Basic Characteristics of the Study Population

A total of 17,640 children and adolescents participated in KiGGS. CAPI data was available for 17,450 children and adolescents (51.3% males and 48.7% females). In Table 1 basic characteristics of the study population are presented. Of the participants 2580 (17.2%) had a two-sided migrant background, 1283 (8.3%) had a one-sided migrant background. The largest ethnic group within KiGGS were Turkish children and adolescents (5.8%; *n* = 874), followed by those from the former Soviet Union (4.4%; *n* = 677) and those from Poland (2.5%; *n* = 372). 20.3% of the children and adolescents with migrant background in KiGGS were not born in Germany.

The distribution of participants by sex was similar among participants with and without migrant backgrounds; merely children and adolescents with migrant background were slightly younger. Clear differences were observed in terms of socio-economic status and living environment. Children and adolescents with migrant backgrounds lived more often in families with low socio-economic status and we found a marked town-country differential, as children and adolescents with migrant background lived more often in urban than in rural areas (Table 1).

### 3.2. Main Findings

#### 3.2.1. Atopic Diseases

We observed a significantly lower prevalence of atopic dermatitis in children and adolescents with a two-sided migrant background for physician-diagnosed lifetime prevalence (8.0%, 7.0–9.1 *vs.* 14.4%, 13.6–15.3) as well as for 12-month prevalence (4.6%, 3.8–5.6 *vs.* 7.7%, 7.1–8.3) compared to non-migrants (Table 2). We did not observe general differences among children and adolescents with migrant background compared to non-migrants for both ever and current asthma and allergic rhinoconjunctivitis, although the prevalences tended to be lower among participants with a two-sided migrant background (Table 2).

The prevalence of atopic diseases tended to increase with the length of stay of the families. For example lifetime prevalence of atopic dermatitis increased among participants with a two-sided migrant background with the length of stay of the families (0–5 years: 5.7%, 3.7–8.7; 6–10 years: 8.2%, 6.2–10.9; 11–15 years: 8.3%, 6.5–10.5; 16–20 years: 6.1%, 4.0–9.3; >20 years: 10.6%, 8.3–13.5). The same holds true for asthma (0–5 years: 3.2%, 1.6–6.2; 6–10 years: 3.3%, 2.0–5.3; 11–15 years: 4.3%, 2.9–6.3; 16–20 years: 5.9%, 3.5–9.6; >20 years: 4.8%, 3.2–7.1) and allergic rhinoconjunctivitis (0–5 years: 5.2%, 3.4–8.1; 6–10 years: 7.1%, 5.2–9.7; 11–15 years: 10.1%, 7.9–12.8; 16–20 years: 15.1%, 11.1–20.2; >20 years: 11.0%, 8.3–14.5) in participants with a two-sided migrant background.

**Figure 1:**
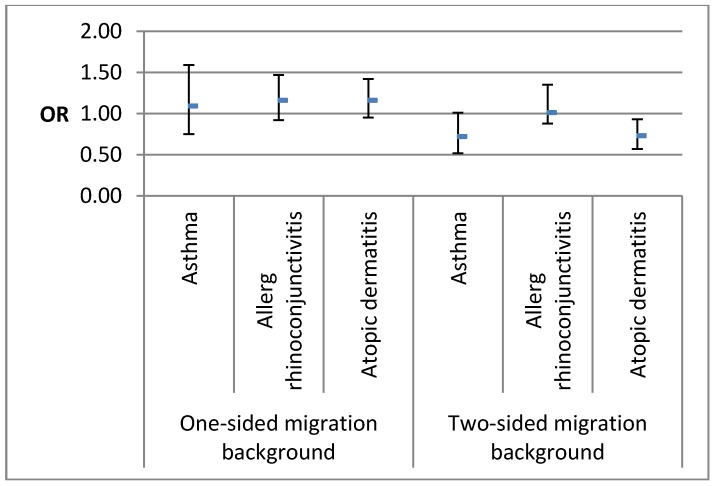
Association between 12-month prevalence of atopic diseases and migrant background. Multivariate models were adjusted for sex, age, socio-economic status, mold-infested rooms, smoking mother and/or father, living environment, birth order and parental atopy.

Associations between atopic diseases in the last 12 months and migrant background were investigated in multiple logistic regression models in order to find out whether migrant background is an independent determinant for the diseases. The results show that children and adolescents with a two-sided migrant background had about one-third less often the chance of having atopic dermatitis (OR 0.73, 0.57–0.93) (Figure 1), independent of sex, age, socio-economic status, mold-infested rooms, smoking mother and/or father, living environment, birth order and parental atopy. On the other hand, we found asthma to be about one-third less likely in children and adolescents with a two-sided migrant background as compared to those with no migrant background, but the association was not statistically significant (OR 0.72, 0.52–1.01). Moreover, we did not find statistically significant differences for any of the atopic diseases in children and adolescents with a one-sided migrant background compared to non-migrants (Figure 1).

#### 3.2.2. Allergic Sensitization

Blood serum samples tested for specific IgE-antibodies were available for 13,100 children and adolescents aged 3–17 years (Table 3). The results show that children and adolescents with a two-sided migrant background were statistically significant less often sensitized to almost all of the allergens tested, except for house dust mites (*Dermatophagoides pteronyssinus* and *Dermatophagoides farinae*), mold (*Cladosporium herbarum* and *Aspergillus fumigatus*) and egg white than non-migrants. For cow’s milk protein, those with a two-sided migrant background were more often sensitized compared to non-migrants (7.1%, 6.0–8.4 *vs.* 5.3%, 4.8–5.9) (Table 3).

In additional analyses we investigated the associations of allergic sensitizations and migrant background in multiple logistic regression models adjusted for age, sex, socio-economic status, mold-infested rooms, smoking mother and/or father, living environment, birth order and parental atopy. Finally, statistically significant associations between allergic sensitizations and a two-sided migrant background remained for birch (OR 0.73, 0.58–0.90), soybean (OR 0.72, 0.54–0.96), peanut (OR 0.69, 0.53–0.90), rice (OR 0.64, 0.48–0.87), potato (OR 0.64, 0.48–0.85), and horse dander (OR 0.58, 0.40–0.85). We did not find statistically significant differences for any of the allergic sensitizations in participants with a one-sided migrant background compared to non-migrants.

## 4. Discussion

In multivariate analyses with substantial adjustment we found atopic dermatitis about one-third less often in children and adolescents with a two-sided migrant background. Almost half of the different allergic sensitizations between non-migrants and children and adolescents with a two-sided migrant background remained statistically significant after adjustment. However, a clear trend in terms of aeroallergen, food allergen or animal allergen sensitization could not be observed. A comparison of sensitization profiles between different epidemiological studies is limited due to widely divergent approaches and differences in number and types of allergens tested [16,17,18].

The findings regarding atopic dermatitis are in line with several other studies on a national level, mainly referring to Turkish children and adolescents living in Germany [19,20,21]. The findings for asthma and allergic rhinoconjunctivitis for a two-sided migrant background point in the same direction as the results from these studies. Another study conducted with data from KiGGS investigated determinants of atopic dermatitis and found that a diagnosis of atopic dermatitis (lifetime prevalence) was less likely in children and adolescents with a two-sided migrant background. Determinants of the other atopic diseases or allergic sensitization to specific IgE antibodies were not investigated [22]. Compared with non-migrants further studies from Italy [23,24], Israel [25] and Australia [26] showed a lower prevalence of allergic diseases among migrants.

Although the results of an analysis of the concept of duration of stay are very interesting and could help to understand a possible association and mechanisms, it could only briefly glossed over in our study. Only 20.3% of the survey participants in KiGGS were not born in Germany. However, analyses of an US population-based sample found that children born outside the United States compared to those born in the United States had significantly lower odds of any atopic disease that increased after residing in the United States for one decade [27]. This was also demonstrated in other studies, including analyses of the Third National Health and Nutrition Examination Survey (NHANESIII) and National Health Interview Survey (NHIS) [28,29,30]. A study from Australia revealed increased asthma symptoms for adolescents who migrated to Australia compared to their peers in their country of origin. Most recent arrivals were less often affected by wheeze (17%), followed by adolescents who lived more than two years in the host country (20%) and highest for those who lived their whole life in Australia (36%) [31]. Similar results were found in an Italian study, where 84% of migrants reported to have no symptoms of allergy and asthma prior to migration [23]. It should, however, be noted that the operationalization of duration of stay does not reflect the real duration of stay of each individual but of the families [26,32,33]. As in KiGGS most of the surveyed children and adolescents did not migrate themselves (second or third generation migrants), this suggests that the observed differences are caused by other influencing factors, *i.e.*, diet, cultural habits and/or by different residential environment and might be associated with growing up in large families or areas with many children as compared to small families (industrialized countries). However, it is possible that families might have spent time living in other “westernized” countries before residing in Germany; information that was not captured by our data. Besides changes in socio-economic position and lifestyle habits, migration is associated with exposure to a new composition of pollutants and allergens, leading to the assumption that the prevalence of atopic diseases among migrants rises with the duration of stay [34]. In line with that, a review by Rottem *et al*. showed a lower prevalence of atopy in underdeveloped countries compared to industrialized countries. This states migration to industrialized countries (allergy-prevalent) causes more allergies and asthma in migrants due to factors that are associated with a longer duration of stay or being born in industrialized countries (second, third generation) compared to those with no migrant background [32].

The observed differences between children and adolescents with a two-sided migrant background compared to non-migrants could be partially an expression of differences in health behavior/health beliefs and/or the utilization of the health care system, difficulties in comprehension or a socio-culturally influenced perception of disease between both groups. Research from different countries, including Germany, has shown that compared to the majority population, migrants generally have less access to health services and health information mainly due to language and/or cultural barriers [35,36,37,38]. Therefore, there might be an underreporting of atopic diseases due to language barriers and difficulties in comprehension among children and adolescents with migrant background (due to differences in perception of disease and clinical symptoms by socio-cultural background) [39,40]. One possible reason for not finding statistically significant differences in participants with a one-sided migrant background is that if one parent does not have any migrant background, the family might be more likely to use the health care system like families without any migrant background. Thus, there might be less underreporting of atopic diseases in participants with a one-sided migrant background. However the observed lower sensitization profiles in migrants diminishes that assumption.

Having a two-sided migrant background was generally associated with low socio-economic status but the associations of SES and migration with atopic dermatitis were independent from each other, as also shown by a more recent analysis based on the KiGGS survey [41]. This probably indicates that the social environment of children and adolescents with and without migrant background differs independently of socio-economic position and implies that there might be further unknown factors that influence the development of atopic diseases. Family history of allergies (at least one atopic disease of mother and/or father) is amongst the strongest determinants of being diagnosed with atopic diseases. It is well known that genetic predisposition plays a crucial role for developing atopic diseases. Thus, we considered family history of allergies in our multivariate analyses and in the result the association of atopic dermatitis and migrant background was independent from family history of allergies.

One possible reason behind the observed differences between migrants and non-migrants can be found in the so called “hygiene hypothesis”, which dates back to 1989 and states that lack of exposure to allergens from the environment (e.g., infectious agents, parasites, viruses, bacteria) early in life results in increased susceptibility of allergic diseases due to reduced “training” of the immune system [42]. Children and adolescents with migrant background might have more often had childhood infections or were more often exposed to infectious agents, parasites *etc*. due to larger families and/or differences in lifestyle and living environment. In line with the described protective sibling effect by Karmaus *et al*. [43], Schmitz *et al*. also found a protective effect for last- and middle-born children regarding allergic rhiniconjunctivitis [41]. However, studies examining duration of stay do not support this mechanism.

### Strengths and Limitations

The main strength of KiGGS is the nationally representative large sample of children and adolescents aged 0–17 years. A non-response analysis did show only little variation between responders and non-responders; information of 89% of the gross sample was available. Therefore, we can assume that our findings are not substantially influenced by selection bias. For the first time, a migrant-specific approach was used that included an oversampling of children and adolescents with migrant background, translation of study information and questionnaires as well as a multiple stage model to include those with language barriers.

A limitation is that the size of the different migrant populations included is heterogeneous and therefore, it is not possible to draw sound conclusions for all migrants in general. A further limitation is that important aspects associated with migration such as health care access, public health literacy, social support after moving into new countries, and changes in lifestyle, which might potentially impact the prevalence of allergic diseases, could not be considered in our analyses.

## 5. Conclusions

We found lower prevalence of atopic dermatitis and allergic sensitization among participants with a two-sided migrant background compared to non-migrants. However, reasons are unclear. Presumably other factors than the known ones (e.g., infections, family history) may have a much stronger effect on the etiology of atopic diseases. Factors other than genetic predisposition, such as environmental factors and living conditions need to be investigated in future research regarding existing differences between migrants and non-migrants as well as associated risk and protective factors in children and adolescents with migrant background. Further studies should also investigate the timing of IgE-testing and duration of stay in Germany as well as first generation migrants compared to second and third generation migrants. This could help to disentangle reasons of the observed lower sensitizations to some of the allergens tested in migrants.

## Figures and Tables

**Table 1:** Basic characteristics of the total study population and stratified by migrant background.

	Total	Migrant Background		
Basic Characteristics	*n* = 17,450 % (n) *	Two-Sided *n* = 2580 % (n) *	One-Sided *n* = 1283 % (n) *	None *n* = 13,507 % (n) *
Sex				
Male	51.3 (8880)	52.1 (1350)	52.5 (656)	51.0 (6842)
Female	48.7 (8570)	47.9 (1230)	47.5 (627)	49.0 (6665)
Age group (in years)				
0–2	13.6 (2770)	12.7 (360)	19.9 (279)	13.0 (2113)
3–6	21.0 (3832)	21.5 (565)	24.2 (315)	20.5 (2920)
7–10	21.7 (4107)	21.3 (606)	21.8 (306)	21.8 (3169)
11–13	17.3 (3040)	18.9 (486)	14.8 (185)	17.3 (2369)
14–17	26.3 (3701)	25.6 (563)	19.4 (198)	27.4 (2936)
Socio-economic status				
High	27.1 (4366)	10.0 (217)	30.3 (376)	30.2 (3143)
Middle	45.4 (7901)	36.3 (834)	42.7 (538)	47.6 (6507)
Low	27.5 (4760)	53.7 (1250)	27.0 (356)	22.1 (3764)
Living environment				
Rural	45.3 (8463)	24.2 (631)	31.3 (429)	51.7 (7365)
Urban	54.7 (8987)	75.8 (1949)	68.7 (854)	48.3 (6142)
Parental atopy				
Yes	37.9 (7341)	25.1 (579)	39.5 (487)	40.5 (5174)
No	62.1 (9372)	74.9 (1772)	60.5 (792)	59.5 (8212)
Mold-infested rooms				
Yes	5.1 (893)	9.9 (245)	5.4 (72)	4.1 (575)
No	94.9 (16,199)	90.1 (2100)	94.6 (1204)	95.9 (12,853)
Smoking mother and/or father				
Yes	49.8 (8716)	54.6 (1380)	53.2 (692)	48.3 (6617)
No	50.2 (8479)	45.4 (1121)	46.8 (692)	51.7 (6758)
Birth order				
Last or middle born	49.8 (8156)	54.2 (1159)	44.2 (552)	49.5 (6441)
First born, as part of multiples or w/o siblings	50.2 (8291)	45.8 (972)	55.8 (687)	50.5 (6629)

* % = weighted sample, n = unweighted; Columns for migration status do not add up to the total n, because of missing values during operationalization of migrant background. 17,450 participants completed the CAPI.

**Table 2:** Lifetime and 12-month prevalence of atopic diseases by migrant background.

Atopic Disease	Migrant Background					
	None		One-Sided		Two-Sided	
	% (95%-CI) *	N **	% (95%-CI) *	N **	% (95%-CI) *	N **
**Lifetime prevalence**						
Asthma	4.7 (4.3–5.1)	593	5.9 (4.6–7.4)	70	4.4 (3.7–5.2)	107
Allergic rhinoconjunctivitis	11.0 (10.3–11.7)	1339	11.1 (9.3–13.2)	129	9.6 (8.3–10.9)	232
Atopic dermatitis	**14.4 (13.6–15.3)**	1920	13.4 (11.6–15.3)	174	**8.0 (7.0–9.1)**	210
					*p* ≤ 0.0001	
**12-month prevalence**						
Asthma	3.1 (2.7–3.4)	381	3.5 (2.6–4.7)	42	2.4 (1.9–3.1)	61
Allergic rhinoconjunctivitis	9.0 (8.4–9.6)	1101	9.2 (7.6–11.0)	104	7.7 (6.8–8.8)	189
Atopic dermatitis	**7.7 (7.1–8.3)**	1069	8.6 (7.3–10.3)	113	**4.6 (3.8–5.6)**	121
					*p* ≤ 0.0001	

* % = weighted sample, ** N = unweighted (Number entries given in bold indicate a significant difference).

**Table 3:** Allergen specific sensitization profiles to 20 common allergens in 3- to 17-year-old children and adolescents (stratified by migrant background).

*n* = 13,100			Migrant Background				Total	
	None		One-Sided		Two-Sided			
	% (95%-CI) *	N **	% (95%-CI) *	N **	% (95%-CI) *	N **	% (95%-CI) *	N **
Sensitized to at least one of the test allergens	40.3 (38.9–41.7)	4006	42.9 (38.9–47.0)	369	39.1 (36.9–41.4)	735	40.3 (38.9–41.5)	5110
*Cladosporium herbarum* (mold)	1.8 (1.5–2.1)	198	2.5 (1.7–3.9)	22	1.2 (0.8–1.9)	22	1.7 (1.5–2.0)	245
*Aspergillus fumigatus* (mold)	2.3 (1.9–2.6)	255	2.6 (1.8–3.8)	24	2.4 (1.8–3.3)	46	2.3 (2.0–2.7)	327
Egg white (food)	4.7 (4.2–5.2)	472	6.6 (5.0–8.8)	56	5.5 (4.5–6.7)	106	5.0 (4.5–5.5)	637
Cow’s milk protein(food)	5.3 (4.8–5.9)	523	6.8 (5.0–9.1)	59	7.1 (6.0–8.4)	138	5.8 (5.2–6.3)	725
Soybean (food)	7.0 (6.2–7.9)	684	5.0 (3.7–6.7)	43	4.0 (3.1–5.2)	77	6.3 (5.6–7.1)	806
Rice (food)	8.0 (7.1–9.0)	794	5.5 (4.1–7.4)	47	4.2 (3.3–5.5)	86	7.2 (6.4–8.0)	930
Potato (food)	9.3 (8.3–10.4)	900	7.3 (5.5–9.7)	59	5.2 (4.1–6.5)	98	8.4 (7.5–9.3)	1059
Apple (food)	9.9 (9.0–10.9)	964	9.5 (7.6–11.9)	80	6.1 (4.9–7.5)	119	9.2 (8.5–10.0)	1167
Carrot (food)	10.4 (9.5–11.4)	1014	9.6 (7.6–12.0)	80	6.6 (5.4–8.0)	129	9.7 (8.9–10.5)	1228
Wheat (food)	10.6 (9.6–11.6)	1050	8.6 (6.6–11.0)	71	7.4 (6.1–8.9)	141	9.9 (9.0–10.7)	1266
Peanut (food)	11.6 (10.5–12.7)	1150	9.7 (7.8–12.0)	81	7.0 (5.8–8.5)	134	10.6 (9.7–11.6)	1369
Mugwort (pollen)	11.6 (10.7–12.6)	1199	10 (8.0–12.4)	85	8.0 (6.6–9.6)	152	10.8 (10.0–11.7)	1440
Common silver birch (pollen)	14.9 (13.9–16.0)	1483	15.9 (13.5–18.6)	131	9.7 (8.4–11.3)	184	14.1 (13.2–15.0)	1806
Cultivated rye (pollen)	22.0 (20.6–23.4)	2175	21.7 (18.7–25.0)	182	17.4 (15.6–19.5)	334	21.1 (20.0–22.3)	2701
Timothy grass (pollen)	23.4 (22.1–24.8)	2322	23.3 (20.1–26.9)	199	19.4 (17.5–21.5)	365	22.7 (21.5–23.9)	2899
Horse dander (animal)	4.8 (4.3–5.3)	476	4.7 (3.4–6.6)	40	2.5 (2.0–3.2)	51	4.4 (4.0–4.8)	569
Cat dander (animal)	8.5 (7.9–9.2)	894	8.6 (6.7–11.1)	71	6.1 (5.0–7.4)	71	8.1 (7.6–8.7)	1088
Dog dander (animal)	10.2 (9.5–11.0)	1022	8.3 (6.5–10.5)	72	7.7 (6.4–9.2)	149	9.6 (9.1–10.3)	1249
*Dermatophagoides farina* (house dust mite)	21.0 (20.1–22.0)	2077	19.4 (16.5–22.7)	165	20.0 (18.1–22.0)	373	20.7 (19.9–21.6)	2627
*Dermatophagoides pteronyssinus* (house dust mite)	21.0 (20.1–21.9)	2085	19.7 (16.6–23.2)	170	21.1 (19.3–23.0)	395	20.9 (20.1–21.8)	2661

* % = weighted sample, ** N = unweighted (Number entries given in bold indicate a significant difference).

## Abbreviations

The following abbreviations are used in this manuscript:

CAPI: Computer Assisted Personal Interview
KiGGS: German Health Interview and Examination Survey for children and adolescents
NHANESIII: Third National Health and Nutrition Examination Survey
NHIS: National Health Interview Survey
OR: Odds Ratio
RKI: Robert Koch Institute
WHO: World Health Organization

## References

[1] Galassi C., De Sario M., Biggeri A., Bisanti L., Chellini E., Ciccone G., Petronio M.G., Piffer S., Sestini P., Rusconi F. (2006). Changes in prevalence of asthma and allergies among children and adolescents in Italy: 1994–2002. Pediatrics.

[2] Horii K.A., Simon S.D., Liu D.Y., Sharma V. (2007). Atopic dermatitis in children in the United States, 1997–2004: Visit trends, patient and provider characteristics, and prescribing patterns. Pediatrics.

[3] Grize L., Gassner M., Wüthrich B., Bringolf-Isler B., Takken-Sahli K., Sennhauser F., Stricker T., Eigenmann P., Braun-Fahrländer C. (2006). Trends in prevalence of asthma, allergic rhinitis and atopic dermatitis in 5–7-year old Swiss children from 1992 to 2001. Allergy.

[4] Mortz C., Lauritsen J., Bindslev-Jensen C., Andersen K.E. (2001). Prevalence of atopic dermatitis, asthma, allergic rhinitis, and hand and contact dermatitis in adolescents. The Odense Adolescence Cohort Study on Atopic Diseases and Dermatitis. Br. J. Dermatol..

[5] Visness C.M., London S.J., Daniels J.L., Kaufman J.S., Yeatts K.B., Siega-Riz A.M., Calatroni A., Zeldin D.C. (2010). Association of childhood obesity with atopic and nonatopic asthma: Results from the National Health and Nutrition Examination Survey 1999–2006. J. Asthma.

[6] Wuthrich B., Schmid-Grendelmeier P. (2003). The atopic eczema/dermatitis syndrome. Epidemiology, natural course, and immunology of the IgE-associated (“extrinsic”) and the nonallergic (“intrinsic”) AEDS. J. Investig. Allergol. Clin. Immunol..

[7] Schernhammer E.S., Vutuc C., Waldhor T., Haidinger G. (2008). Time trends of the prevalence of asthma and allergic disease in Austrian children. Pediatr. Allergy Immunol..

[8] Kjaer H.F., Eller E., Host A., Andersen K.E., Bindslev-Jensen C. (2008). The prevalence of allergic diseases in an unselected group of 6-year-old children. The DARC birth cohort study. Pediatr. Allergy. Immunol..

[9] Hurrelmann K. (2003). Jugendgesundheitssurvey: Internationale Vergleichsstudie im Auftrag der Weltgesundheitsorganisation WHO.

[10] Kamtsiuris P., Lange M., Schaffrath R.A. (2006). The German Health Interview and Examination Survey for Children and Adolescents (KiGGS): Sample design, response and nonresponse analysis. Bundesgesundheitsblatt Gesundheitsforschung Gesundheitsschutz.

[11] Kurth B.-M., Kamtsiuris P., Hölling H., Schlaud M., Dölle R., Ellert U., Kahl H., Knopf H., Lange M., Mensink G.B. (2008). The challenge of comprehensively mapping children’s health in a nation-wide health survey: Design of the German KiGGS-Study. BMC. Public Health.

[12] Holling H., Kamtsiuris P., Lange M., Thierfelder W., Thamm M., Schlack R. (2007). The German Health Interview and Examination Survey for Children and Adolescents (KiGGS): Study management and conduct of fieldwork. Bundesgesundheitsblatt Gesundheitsforschung Gesundheitsschutz.

[13] Schenk L., Ellert U., Neuhauser H. (2007). Children and adolescents in Germany with a migration background. Methodical aspects in the German Health Interview and Examination Survey for Children and Adolescents (KiGGS). Bundesgesundheitsblatt Gesundheitsforschung Gesundheitsschutz.

[14] Lange M., Kamtsiuris P., Lange C., Schaffrath Rosario A., Stolzenberg H., Lampert T. (2007). Sociodemographic characteristics in the German Health Interview and Examination Survey for Children and Adolescents (KiGGS)—Operationalisation and public health significance, taking as an example the assessment of general state of health. Bundesgesundheitsblatt Gesundheitsforschung Gesundheitsschutz.

[15] Winkler J., Stolzenberg H. (1999). Der Sozialschichtindex im Bundes-Gesundheitssurvey. Gesundheitswesen.

[16] Kucukosmanoglu E., Yazi D., Yesil O., Akkoc T., Gezer M., Ozdemir C., Bakirci N., Bahceciler N.N., Barlan I.B. (2008). Prevalence of immediate hypersensitivity reactions to cow’s milk in infants based on skin prick test and questionnaire. Allergol. Immunopathol..

[17] Jarvinen K.M., Suomalainen H. (2001). Development of cow’s milk allergy in breast-fed infants. Clin. Exp. Allergy.

[18] Eggesbo M., Botten G., Halvorsen R., Magnus P. (2001). The prevalence of CMA/CMPI in young children: The validity of parentally perceived reactions in a population-based study. Allergy.

[19] Grüber C., Meinlschmidt G., Bergmann R., Wahn U., Stark K. (2002). Is early BCG vaccination associated with less atopic disease? An epidemiological study in German preschool children with different ethnic backgrounds. Pediatr. Allergy Immunol..

[20] Grüber C., Illi S., Plieth A., Sommerfeld C., Wahn U. (2002). Cultural adaptation is associated with atopy and wheezing among children of Turkish origin living in Germany. Clin. Exp. Allergy.

[21] Kabesch M., Schaal W., Nicolai T., Von Mutius E. (1999). Lower prevalence of asthma and atopy in Turkish children living in Germany. Eur. Respir. J..

[22] Apfelbacher C.J., Diepgen T.L., Schmitt J. (2011). Determinants of eczema: Population-based cross-sectional study in Germany. Allergy.

[23] Tedeschi A., Barcella M., Bo G.A., Miadonna A. (2003). Onset of allergy and asthma symptoms in extra-European immigrants to Milan, Italy: Possible role of environmental factors. Clin. Exp. Allergy.

[24] Marcon A., Cazzoletti L., Rava M., Gisondi P., Pironi V., Ricci P., De Marco R. (2011). Incidence of respiratory and allergic symptoms in Italian and immigrant children. Respir. Med..

[25] Farfel A., Green M.S., Shochat T., Noyman I., Levy Y., Afek A. (2007). Trends in specific morbidity prevalence in male adolescents in Israel over a 50 year period and the impact of recent immigration. Isr. Med. Assoc. J..

[26] Leung R.C., Carlin J.B., Burdon J.G., Czarny D. (1994). Asthma, allergy and atopy in Asian immigrants in Melbourne. Med. J. Aust..

[27] Silverberg J.I., Simpson E.L., Durkin H.G., Joks R. (2013). Prevalence of allergic disease in foreign-born American children. JAMA Pediatr..

[28] Holguin F., Mannino D.M., Anto J., Mott J., Ford E.S., Teague W.G., Redd S.C., Romieu I. (2005). Country of birth as a risk factor for asthma among Mexican Americans. Am. J. Respir. Crit. Care. Med..

[29] Eldeirawi K., McConnell R., Freels S., Persky V.W. (2005). Associations of place of birth with asthma and wheezing in Mexican American children. J. Allergy. Clin. Immunol..

[30] Brugge D., Lee A.C., Woodin M., Rioux C. (2007). Native and foreign born as predictors of pediatric asthma in an Asian immigrant population: A cross sectional survey. Environ. Health.

[31] Gibson P.G., Henry R.L., Shah S., Powell H., Wang H. (2003). Migration to a western country increases asthma symptoms but not eosinophilic airway inflammation. Pediatr. Pulmonol..

[32] Rottem M., Szyper-Kravitz M., Shoenfeld Y. (2005). Atopy and asthma in migrants. Int. Arch. Allergy. Immunol..

[33] Leung R. (1996). Asthma and migration. Respirology.

[34] Coultas D.B., Gong H., Grad R., Handler A., McCurdy S.A., Player R., Rhoades E.R., Samet J.M., Thomas A., Westley M. (1994). Respiratory diseases in minorities of the United States. Am. J. Respir. Crit. Care. Med..

[35] Spallek J., Zeeb H., Razum O. (2010). Prevention among immigrants: The example of Germany. BMC Public Health.

[36] Khan N.A., Saboor H.T., Qayyum Z., Khan I., Habib Z., Waheed H.T. (2013). Barriers to accessing the German health-care system for Pakistani immigrants in Berlin, Germany: A qualitative exploratory study. Lancet.

[37] Henderson S., Kendall E. (2011). Culturally and linguistically diverse peoples’ knowledge of accessibility and utilisation of health services: Exploring the need for improvement in health service delivery. Aust. J. Prim. Health.

[38] Povlsen L., Olsen B., Ladelund S. (2005). Diabetes in children and adolescents from ethnic minorities: Barriers to education, treatment and good metabolic control. J. Adv. Nurs..

[39] Harris J.M., Cullinan P., Williams H., Mills P., Moffat S., White C., Newman T.A. (2001). Environmental associations with eczema in early life. Br. J. Dermatol..

[40] Levy R.M., Gelfand J.M., Yan A.C. (2003). The epidemiology of atopic dermatitis. Clin. Dermatol..

[41] Schmitz R., Atzpodien K., Schlaud M. (2012). Prevalence and risk factors of atopic diseases in German children and adolescents. Pediatr. Allergy. Immunol..

[42] Strachan D.P. (1989). Hay fever, hygiene, and household size. BMJ.

[43] Karmaus W., Botezan C. (2002). Does a higher number of siblings protect against the development of allergy and asthma? A review. J. Epidemiol. Community Health.

